# Supplementary material for: SRSF3 knockdown-induced cellular senescence as a possible therapeutic strategy for non-small cell lung cancer
Source: bioRxiv. 2025 May 9:2025.05.05.652234. Preprint. [Version 1] doi: 10.1101/2025.05.05.652234 (PMC12190769; doi:10.1101/2025.05.05.652234)

bioRxiv preprint doi: https://doi.org/10.1101/2025.05.05.652234; this version posted May 9, 2025. The copyright holder for this preprint (which was not certified by peer review) is the author/funder. This article is a US Government work. It is not subject to copyright under 17 USC 105 and is also made available for use under a CC0 license.



**Supplementary Table 1.** Differentially expressed genes (DEGs) commonly reported in SRSF3 inhibition and in NSCLC development

| Down in SRSF3 inhibition[1]<br>Up in NSCLC[2] | Up in SRSF3 inhibition[1]<br>Down in NSCLC[2] |
|---|---|
| ASPM | MFAP4 |
| UBE2C | IGSF10 |
| RRM2 | DNAAF1 |
| TOP2A | AOC3 |
| IGFBP3 | FOSB |
| | ABI3BP |

[1] Downregulated or upregulated DEGs in SRSF3 inhibition were from Song *et al.* (ref. (13), Supplementary Table S3), which compared wild-type and SRSF3-knocked-out glioma stem-like cells.
[2] Upregulated or downregulated DEGs in NSCLC development were from Wang *et al.* (ref. (19), Supplementary Table S1), which compared NSCLC tissues with matched non-tumor tissues.

bioRxiv preprint doi: https://doi.org/10.1101/2025.05.05.652234; this version posted May 9, 2025. The copyright holder for this preprint (which was not certified by peer review) is the author/funder. This article is a US Government work. It is not subject to copyright under 17 USC 105 and is also made available for use under a CC0 license.



**Supplementary Figure 1.** Poor patient prognosis associated with high levels of expression of TOP2A (**A**), UBE2C (**B**) and ASPM (**C**). The TCGA data of lung adenocarcinoma RNA-sequencing were analyzed for overall survival in high-level group (n=250) versus low-level group (n=251) with the Kaplan-Meier method. HR, hazard ratio.

was not certified by peer review) is the author/funder. This article is a US Government work. It is not subject to copyright under 17 USC 1
and is also made available for use under a CC0 license.

# Supplementary Figure 1

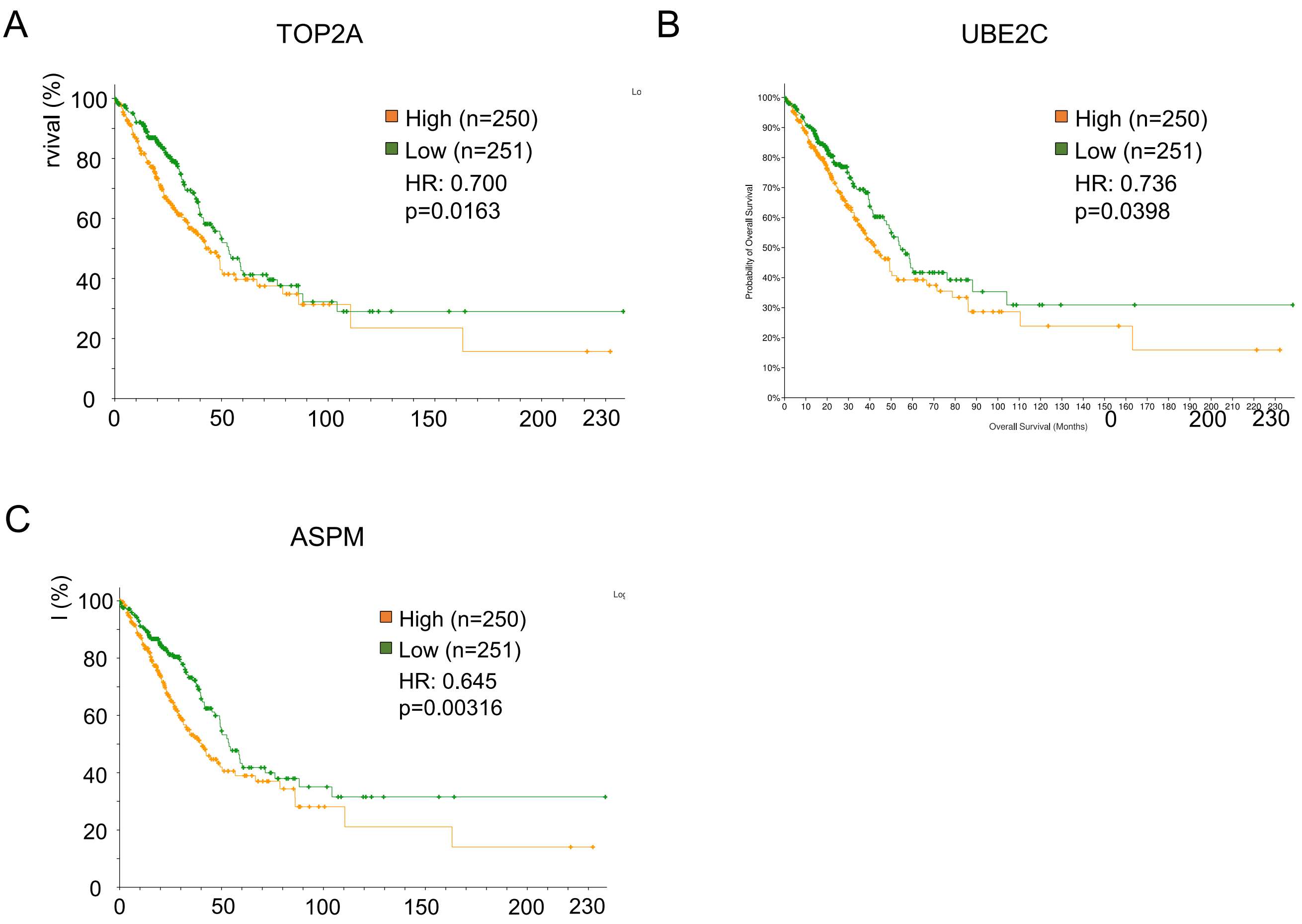

Supplement: 1 [file NIHPP2025.05.05.652234V1-supplement-1.pdf]
